# Femoral component rotational alignment in robotic‐assisted total knee arthroplasty with functional knee positioning varies across knee phenotypes without affecting clinical outcomes

**DOI:** 10.1002/ksa.12732

**Published:** 2025-06-29

**Authors:** Francesco Zambianchi, Gabriele Bazzan, Vincenzo Iorio, Riccardo Cuoghi Costantini, Stefano Seracchioli, Fabio Catani

**Affiliations:** ^1^ Department of Orthopaedics and Traumatology, Azienda Ospedaliero‐Universitaria di Modena University of Modena and Reggio‐Emilia Modena Italy; ^2^ Department of Orthopaedics and Traumatology Ospedale Maggiore di Lodi Lodi Italy; ^3^ Ab Medica S.p.A. Cerro Maggiore Milan Italy; ^4^ Department of Medical and Surgical Sciences for Mother, Child and Adult University of Modena and Reggio‐Emilia Modena Italy; ^5^ Department of Orthopaedic Surgery, School of Medicine University of Pittsburgh Pittsburgh Pennsylvania USA

**Keywords:** femoral component, patient‐reported outcome measures, robotic‐arm assisted, rotational alignment, total knee arthroplasty

## Abstract

**Purpose:**

This retrospective, observational study aimed to investigate the distribution of femoral component rotational alignment among different knee phenotypes and assess the clinical relevance of femoral component rotation variability in patients undergoing image‐based robotic‐assisted total knee arthroplasty (RA‐TKA) performed with functional knee positioning (FP). It was hypothesized that femoral component rotational alignment can change based on preoperative knee phenotypes, without affecting clinical outcomes.

**Methods:**

A total of 256 patients who underwent image‐based RA‐TKA with the Mako robotic system at a single centre from February 2020 to March 2022 were included. Preoperative robotic‐derived computed tomography (CT) data were gathered to determine the Coronal Plane Alignment of the Knee (CPAK) classification and the axial orientation of the distal femur. Intraoperative data relative to femoral component rotational alignment were collected. At a minimum of 24 months post‐operatively, patients were administered the Forgotten Joint Score‐12 (FJS‐12) and the Knee Injury and Osteoarthritis Outcome Score for Joint Replacement (KOOS‐JR). The distribution of femoral component rotation among phenotypes, as well as the relationships between alignment parameters and clinical outcomes, were assessed by means of linear regression models and one‐way analysis of variance.

**Results:**

A total of 219 cruciate retaining TKAs were considered for implant positioning assessment. Femoral component rotational alignment varied significantly among different knee phenotypes, ranging from −6.9° internal to 6.6° external rotation with respect to surgical transepicondylar axis. No significant association was found between femoral component rotation and patient‐reported outcomes (FJS‐12 and KOOS‐JR). Three patients (1.4%) underwent RA‐TKA revision, while no patello‐femoral complications were reported in any of the patients.

**Conclusions:**

In the setting of image‐based RA‐TKA performed with FP, the rotational alignment of the femoral component changes significantly among different knee phenotypes. These variations do not affect clinical outcomes at a minimum of 2 years of follow‐up and do not result in significant patello‐femoral complications.

**Level of Evidence:**

Level III.

**Abbreviations**
3D: three‐dimensional
aHKA: aritmetic hip–knee–ankle angle
AP: antero‐posterior
CI: confidence interval
CPAK: Coronal Plane Alignment of the Knee
CR: cruciate retaining
CT: computed tomography
DAIR: debridement, antibiotic and implant retention
FJS‐12: Forgotten Joint Score‐12
FP: functional knee positioning
JLO: joint line orientation
KOOS‐JR: Knee Injury and Osteoarthritis Outcome Score for Joint Replacement
LDFA: lateral distal femoral angle
MA: mechanical alignment
MPTA: medial proximal tibial angle
PCA: posterior condylar axis
PROM: patient‐reported outcome measure
RA: robotic‐arm assisted
SD: standard deviation
TEA: transepicondylar axis
TKA: total knee arthroplasty

## INTRODUCTION

In total knee arthroplasty (TKA), the orientation of the femoral component is paramount for achieving satisfactory clinical outcomes and survivorship [21]. Historically, following the principles of mechanical alignment (MA), knee flexion balancing was performed systematically and often resorting to soft tissue releases. In particular, the rotational alignment of the femoral component was typically determined using anatomical landmarks such as the posterior condylar axis (PCA), the transepicondylar axis (TEA), and Whiteside's line [33]. Despite being widely utilized, these traditional techniques were subject to some degree of variability and could lead to femoral component rotational malalignment, complicating outcomes with issues like anterior knee pain, knee stiffness, flexion instability and altered kinematics [13, 25].

Recent advancements in the field of robotic and navigated TKA have significantly improved the methodology for achieving knee balance with personalized component alignment. Robotic‐assisted systems offer surgeons real‐time feedback and increased accuracy in the alignment of the tibial and femoral components [36]. Use of image‐based robotic systems coupled with functional knee positioning (FP) provides a three‐dimensional (3D) approach to implant positioning, which optimizes the balancing of both the patello‐femoral and tibio‐femoral joints [19], as well as implementing personalized components' placement and achieving satisfactory clinical outcomes [17, 24]. Several studies have reported on boundaries for femoral and tibial component coronal alignment in FP [5, 10, 17, 30, 35]. However, the impact of rotational changes on flexion balance during robotic‐assisted TKA with FP has been poorly reported and femoral component axial alignment boundaries have not been clearly defined. Although robotic‐assisted (RA)‐TKA with FP offers benefits such as reduced gap imbalance and fewer soft tissue releases [30, 32], there is still limited knowledge about the variability regarding femoral component rotation. Excessive internal rotation of the femoral component raises concerns about patello‐femoral instability and anterior knee pain [6, 13, 25], while increased values of external rotation of the femoral component may lead to an increase in the medial flexion gap. Therefore, the aim of the present study was to investigate the variation of femoral component rotational alignment among different knee phenotypes and assess the clinical relevance of femoral component rotation variability in a consecutive series of patients undergoing cruciate retaining (CR) image‐based RA‐TKA performed with FP. It was hypothesized that femoral component rotational alignment may change based on the preoperative knee phenotypes, without affecting the clinical outcome.

## METHODS

**Figure 1:**
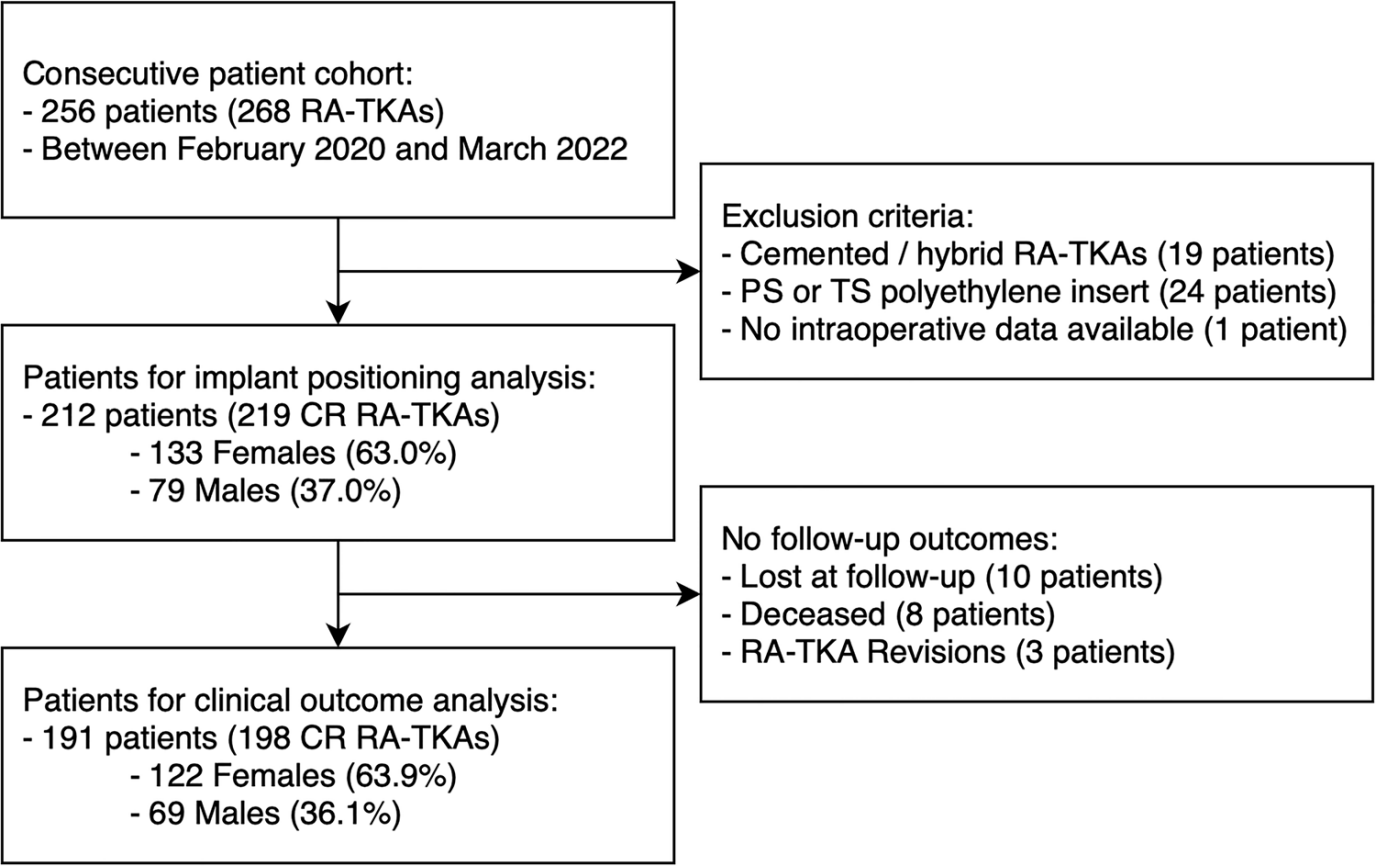
Flow chart of the inclusion process. CR, cruciate retaining; PS, posterior stabilized; RA‐TKA, robotic arm‐assisted total knee arthroplasty; TS, total stabilized.

This was a retrospective, observational study that included a consecutive series of 256 patients who underwent image‐based RA‐TKA at a single centre between February 2020 and March 2022. All procedures were conducted using the Triathlon Tritanium (Stryker) knee design with the Mako robotic system (Mako Surgical Corp. [Stryker]). Patients included in the series had a radiographic diagnosis of primary or post‐traumatic end‐stage knee osteoarthritis and persistent knee pain. Exclusion criteria for CR cementless TKA encompassed inflammatory arthropathies, neurologic disorders, functional incompetence of the posterior cruciate ligament or collateral ligaments, the requirement for higher degrees of constraint and severe osteopenia or osteoporosis, as observed intraoperatively. After applying the exclusion criteria, a total of 212 subjects (219 knees) were included for study assessment (Figure 1).

Preoperative and intraoperative robotic‐derived computed tomography (CT) data were gathered to assess mechanical medial proximal tibial angle (MPTA) and mechanical lateral distal femoral angle (LDFA), femoral axial alignment and tibial posterior slope alignment, as well as the 3D alignment of the implant. The Coronal Plane Alignment of the Knee (CPAK) classification [23] was determined preoperatively based on the robotic‐derived CT data.

### Preoperative procedure

**Figure 2:**
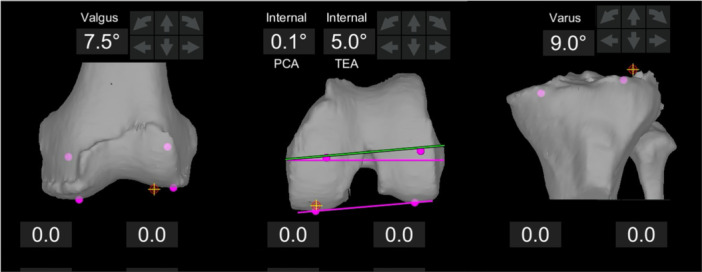
Intraoperative screenshot of the Mako (Mako Surgical Corp. [Stryker]) robotic system, displaying the CT‐based three‐dimensional joint line orientation of the femur and tibia in a left knee. Femoral and tibial coronal alignment, as well as femoral transverse orientation are obtained by equalizing medial and lateral bony resections. In this case, femoral mechanical angle is −7.5° (valgus), tibial mechanical angle is 9.0° (varus). Therefore, LDFA is 90° + (−7.5°) = 82.5° and MPTA is 90° − 9.0° = 81°. Conversely, PCA is in −5.0° (internal) rotation relative to TEA. CT, computed tomography; LDFA, lateral distal femoral angle; MPTA, medial proximal tibial angle; PCA, posterior condylar axis; TEA, transepicondylar axis.

Preoperatively, a CT scan of the hip, knee, and ankle was performed on the operative side. CT axial images were exported, and segmentation and planning were performed using dedicated software for the robotic‐assisted system to produce a 3D model specific to the patient's anatomy. The following reference axes were calculated based on bony landmarks from the preoperative CT scan: (1) femoral mechanical axis, the straight line connecting the centre of the femoral head to the centre of the distal femoral articular surface; (2) surgical TEA, the line connecting the tip of the lateral femoral epicondyle and the medial femoral sulcus and (3) tibial mechanical axis, the axis connecting the midpoint between the anterior cruciate ligament footprint and the tibial spines with the ankle centre, the midpoint between the most prominent point on the medial and lateral malleolus [35]. The joint line orientation of the femur and tibia in the coronal plane was evaluated in relation to their mechanical axes on CT slices. The LDFA consists of the lateral angle measured between the mechanical axis of the femur and the tangent line to the distal femoral condyles. The MPTA is expressed as the medial angle formed by the tibial mechanical axis and the tibial joint line. The coronal alignment of the fetal and tibial components was planned preoperatively by setting equal medial and lateral bone resections, thus matching the preoperative LDFA and MPTA and correcting for bone wear when present [34]. Consequently, the MPTA and LDFA were calculated as follows: LDFA = 90° + femoral joint line axis, as displayed by the robotic software by setting equal distal femoral resections. MPTA = 90° − tibial joint line axis, as displayed by the robotic software by setting equal medial and lateral tibial resections. The rotational alignment of the distal femur was calculated as the angle between TEA and the tangent to the posterior femoral condyles (Figure 2). The posterior tibial slope was defined as the angle formed between the plane perpendicular to the tibial mechanical axis and the line tangent to the most prominent parts of the anterior and posterior cortices of the medial and lateral tibial compartment. Positive values represented varus alignment and external rotation. The coronal alignment of the femoral and tibial components was preoperatively planned by ensuring equal medial and lateral bone resections, thereby aligning with the preoperative LDFA and MPTA, while accounting for any bone wear if present. The coronal alignment of the tibial component was set within a range of 4° varus to 2° valgus (i.e., MPTA 86–92°), and this alignment was not changed during the surgery. The rotational alignment of the femoral component was planned by aligning it with the PCA and ensuring it matched the profile of the femoral trochlear groove, as seen on the preoperative CT scan. In the sagittal plane, femoral component placement was planned to adjust component sizing while preventing anterior notching. The tibial component was aligned with the slope of the unaffected compartment, without exceeding 7°. In the axial plane, it was aligned with the tibial antero‐posterior (AP) axis [1], ensuring adequate medio‐lateral and AP bone coverage. The distal and posterior femoral cuts were planned to maintain the height of the medial femoral joint line in both flexion and extension [35].

### Intraoperative procedure

Femoral and tibial tracker arrays were secured to the patient's femur and tibia. Then, bone registration was performed. The intraoperative strategy for knee balancing adhered to the principles of FP, without tibial precut. Intraoperative adjustments were made to the implant positioning to achieve balanced flexion and extension gaps, aiming for an 18–19 mm medial gap in extension and flexion with a residual laxity of 1–2 mm in the lateral compartment. Femoral component coronal alignment was fine‐tuned within a safe zone of 5° valgus to 5° varus (i.e., LDFA 85–95°), while femoral component rotation, that was initially set parallel to the PCA, was internally or externally rotated to achieve flexion balance, with no rotational boundaries. Bone resections were executed according to the plan, considering joint line height in extension and flexion, and using the haptic‐guided robotic arm. Data on femoral coronal, axial orientation and tibial component coronal and sagittal alignment were routinely collected. Trial prosthetic components were then implanted, and alignment and gaps were verified. Tibial rotational alignment was checked and confirmed aiming for tibio‐femoral congruency in extension. Lateral patellar facetectomy was commonly performed, and the patella was generally not resurfaced. If patellar tracking was suboptimal, the patella was reshaped, and overall thickness was reduced to avoid overstuffing and tilting. Final cementless components were impacted.

### Post‐operative procedure

The radiographic CPAK classification was modified using CT images to facilitate preoperative phenotype categorization into classes. According to the CPAK method, joint line orientation (JLO) was defined as the sum of the MPTA and the LDFA, while the arithmetic hip–knee–ankle angle (aHKA) was calculated as MPTA–LDFA [23]. JLO and aHKA were derived from preoperative CT measurements, as provided by the dedicated robotic‐assisted software [8, 16].

As part of the institution's protocols, patients were followed up with clinical periodic assessment post‐operatively at 3, 6, 12 months and then every year. At a minimum of 24 months post‐operatively, patients were administered the Forgotten Joint Score‐12 (FJS‐12) [4], the Knee Injury and Osteoarthritis Outcome Score for Joint Replacement (KOOS‐JR) [22], and a satisfaction survey using a 5‐level Likert scale, made of 5 items: ‘very satisfied’, ‘satisfied’, ‘neutral’, ‘not satisfied’ and ‘strongly not satisfied’. Post‐operative complications and revisions were recorded. Patients who did not attend follow‐up visits were contacted by telephone; if unresponsive after 3 attempts they were considered lost to follow‐up.

The present study adheres to the principles outlined by the Declaration of Helsinki and follows Good Clinical Practice guidelines for each step performed during the study, including data collection, analysis and reporting. IRB approval was obtained by Comitato Etico Area Vasta Emilia Nord (7/2024/OSS*/AOUMO, transmitted with protocol no. 4527/2024).

### Statistical analysis

Descriptive statistics were computed for analyzed variables overall and by preoperative CPAK class. Means ± standard deviations (SDs) were used to describe numerical variables, whereas absolute and percentage frequencies were used to describe categorical variables. For each numerical variable, a Shapiro–Wilk test was used to test for normality.

One‐way analysis of variance was performed to estimate the overall association between preoperative CPAK class and femoral component rotation, tibial component coronal angle and sagittal alignment, as well as the association between distal femur axial alignment, tibial component coronal angle and sagittal alignment. In addition, linear regressions were modelled to evaluate the estimated difference between each pair of CPAK classes. When pairwise comparing CPAK classes, Bonferroni correction was applied to deal with multiple comparisons. Multivariable linear regression models were fitted to examine the association between tibial coronal, sagittal alignment, and femoral rotation. These models included tibial coronal, sagittal alignment, and femoral component coronal alignment as covariates. Regression slopes were reported, with 95% confidence intervals (CIs) and *p* values. The clinical impact of femoral component rotation was explored by correlating it with patient‐reported outcomes (post‐operative FJS‐12 and KOOS‐JR) using Pearson's product‐moment correlation, and these relationships were visually represented with scatter plots and regression lines, fitted using simple linear regression models.

The significance level alpha was set equal to 0.05. All analyses were carried out using R version 4.3.2 statistical software (The R Foundation for Statistical Computing).

## RESULTS

**Figure 3:**
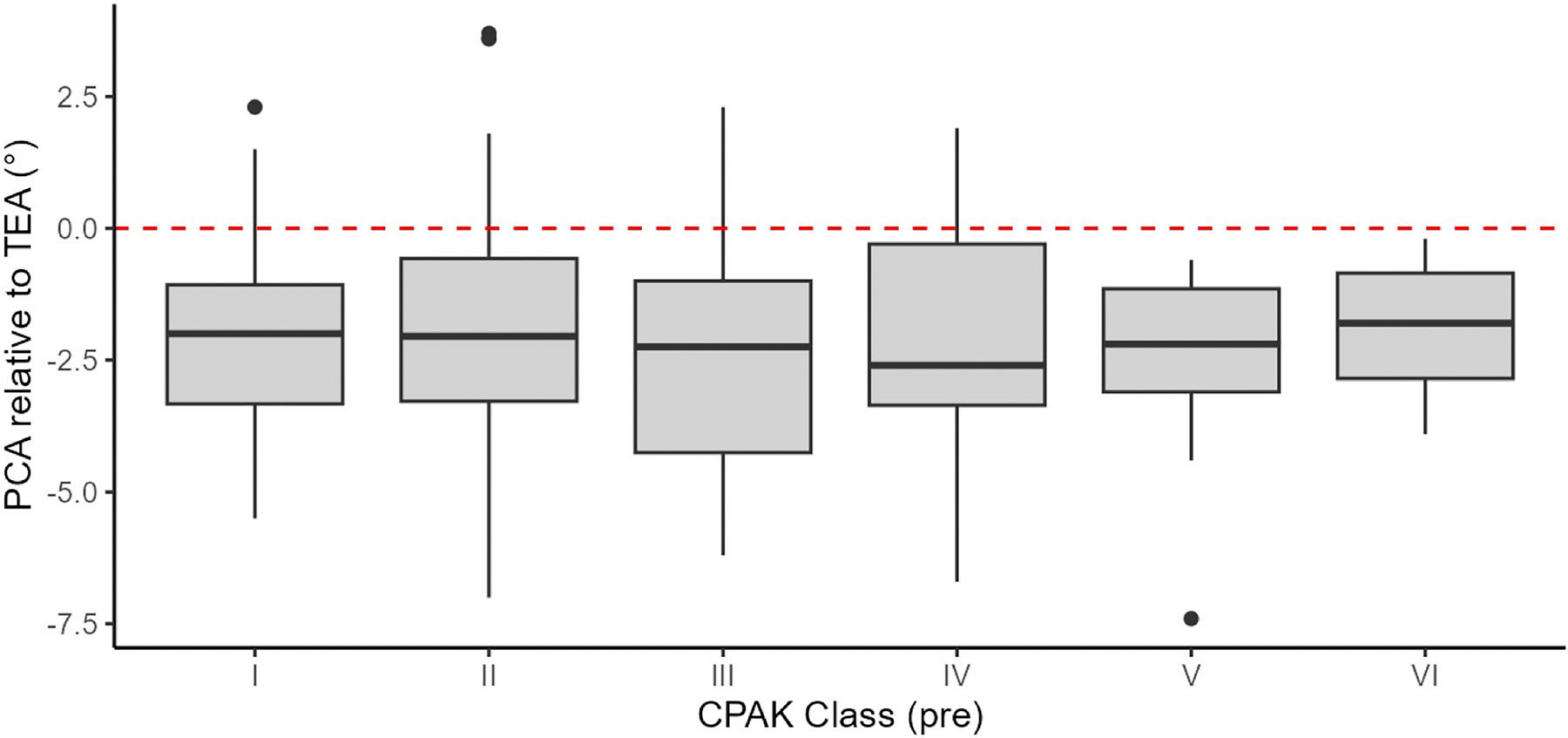
Boxplot showing the distribution of femoral axial alignment, expressed as the angular relationship between PCA and TEA by preoperative CPAK class. Positive values express external rotation. CPAK, Coronal Plane Alignment of the Knee; PCA, posterior condylar axis; TEA, transepicondylar axis.

A total of 219 CR RA‐TKAs were performed on 212 patients, consisting of 133 females (63.0%) and 79 males (37.0%). The mean age of the cohort was 70.3 ± 9.0 years, and the mean body mass index was 29.0 ± 4.4. The mean preoperative aHKA, MPTA and LDFA were 0.9 ± 4.9° varus, 3.4 ± 2.8° and −2.5 ± 3.2°, respectively. On average, the PCA was in internal rotation relative to TEA, with a mean value of −2.1 ± 2.0°. The axial alignment of the distal femur in the osteoarthritic knee population did not vary across different preoperative CPAK phenotypes (p = 0.809) (Table 1, Figure 3).

**Table 1:** Mean femoral axial alignment expressed as the angle by PCA relative to TEA, with respect to preoperative CPAK classes.

Parameter	CPAK class	*N*	Mean ± SD (°)	*p* value	ANOVA
PCA (TEA 0°)	I	68	−2.1 ± 1.7	Reference	0.809
	II	52	−1.9 ± 2.4	0.528	
	III	44	−2.4 ± 2.0	0.480	
	IV	20	−1.9 ± 2.1	0.723	
	V	28	−2.5 ± 1.8	0.519	
	VI	7	−1.9 ± 1.4	0.790	

*Note*: Positive values express external rotation.

Abbreviations: ANOVA, analysis of variance; CPAK, Coronal Plane Alignment of the Knee; PCA, posterior condylar axis; SD, standard deviation; TEA, transepicondylar axis.

### Implant positioning

**Figure 4:**
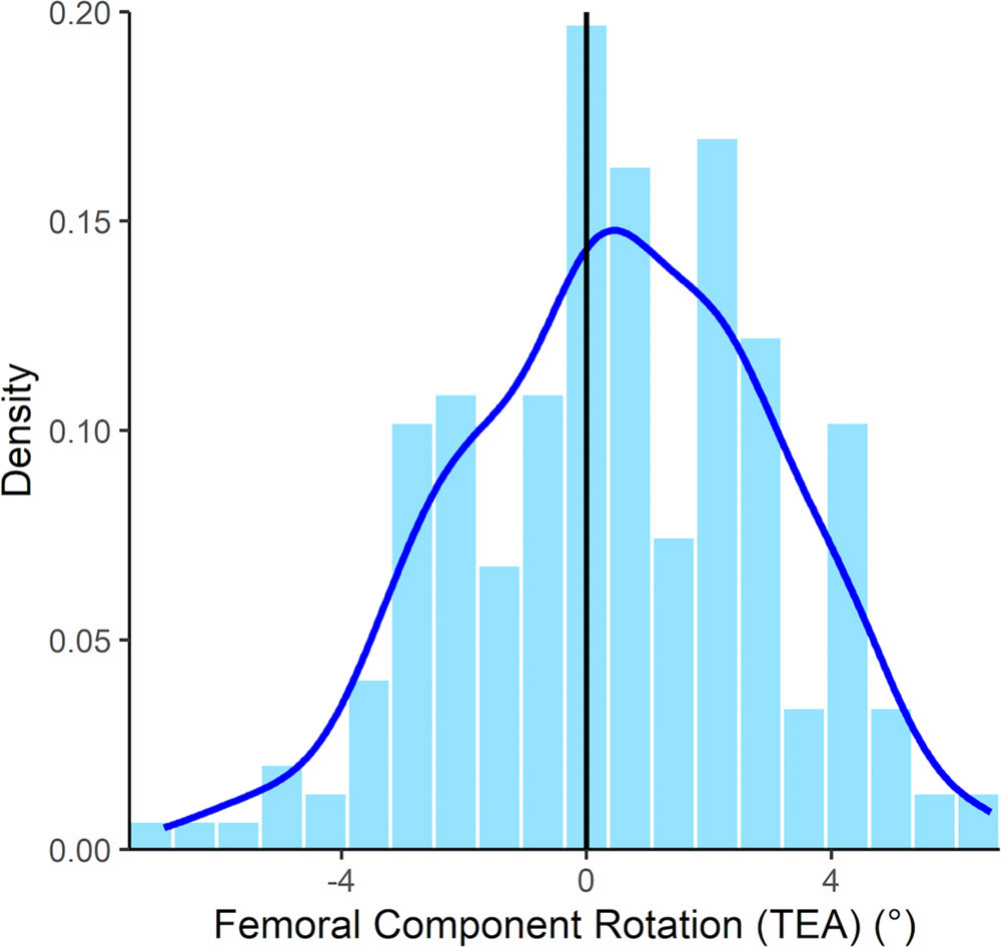
Distribution of femoral component rotational alignment values with reference to TEA. TEA, transepicondylar axis.

**Figure 5:**
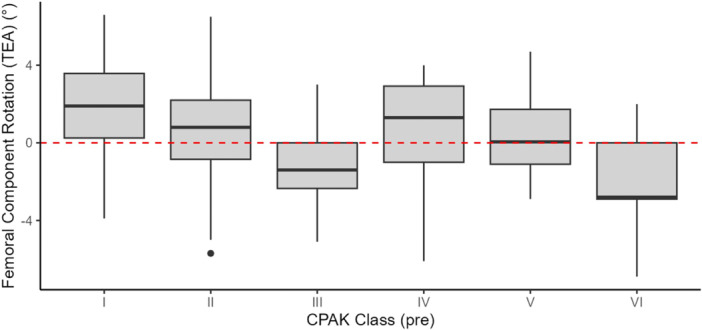
Boxplot showing the distribution of femoral component rotational alignment relative to TEA by preoperative CPAK class. Positive values express external rotation. CPAK, Coronal Plane Alignment of the Knee; PCA, posterior condylar axis; TEA, transepicondylar axis.

The tibial component was, on average, placed at 2.0 ± 1.3° varus and 4.2 ± 1.2° posterior slope. Femoral component rotation was on average in slight external rotation relative to TEA, with a mean value of 0.5 ± 2.6° and ranged from −6.9° (internal) to 6.6° (external) (Figure 4). Rotational alignment of the femoral component changed significantly among the different preoperative phenotypes (*p* < 0.001) (Table 2, Figure 5), except for mean femoral component rotational alignment in CPAK classes I and IV (*p* = 0.086). While significant differences were detected among different preoperative phenotypes for tibial component coronal alignment (*p* < 0.001), no significant differences were observed for the sagittal orientation (*p* = 0.051) (Table 2).

**Table 2:** Femoral component rotational alignment relative to TEA, tibial component coronal and sagittal alignment with respect to preoperative CPAK classes.

Parameter	CPAK class	*N*	Mean ± SD (°)	*p* value	ANOVA
Femoral component rotation (TEA)	I	68	1.8 ± 2.2	Reference	<0.001
	II	52	0.5 ± 2.5	0.026	
	III	44	−1.2 ± 2	<0.001	
	IV	20	0.8 ± 2.7	0.514	
	V	28	0.3 ± 2.2	0.149	
	VI	7	−1.9 ± 2.9	<0.001	
Tibial component coronal alignment	I	68	2.7 ± 0.9	Reference	<0.001
	II	52	2.3 ± 0.9	0.253	
	III	44	0.6 ± 1.2	<0.001	
	IV	20	2.2 ± 0.7	0.230	
	V	28	1.3 ± 1.3	<0.001	
	VI	7	−0.3 ± 0.8	<0.001	
Tibial component sagittal alignment	I	68	4.4 ± 1.2	Reference	0.051
	II	52	4.1 ± 1.1	0.459	
	III	44	3.7 ± 1.2	0.010	
	IV	20	4.3 ± 1.0	1.000	
	V	28	4.0 ± 1.0	1.000	
	VI	7	3.9 ± 1.5	1.000	

*Note*: Positive values express varus alignment and external rotation.

Abbreviations: ANOVA, analysis of variance; CPAK, Coronal Plane Alignment of the Knee; TEA, transepicondylar axis; SD, standard deviation.

A linear, statistically significant association was identified between femoral component rotational alignment and tibial component coronal alignment (regression slope = 0.62; 95% CI: 0.35–0.88; *p* value < 0.001), whereas the relationship between femoral rotation and tibial posterior slope was not statistically significant (regression slope = 0.12; 95% CI: −0.16 to 0.40; *p* value: 0.398).

### Patient‐reported outcome measures (PROMs)

A total of 21 subjects did not have post‐operative PROMs available; hence, 191 subjects (198 CR RA‐TKAs) underwent clinical assessment at a minimum of 24 months post‐operatively (Figure 1). At a mean of 3.1 ± 1.6 years, the mean values of post‐operative FJS‐12, KOOS‐JR and patients' satisfaction were 87.5 ± 15.4, 85.6 ± 18.1 and 4.4 ± 1.1, respectively. No significant association was found between femoral component rotation, FJS‐12 and KOOS‐JR (Table 3). These correlations were also computed separately for each preoperative CPAK class and were found to be very weak or highly variable, suggesting that these outcomes were not affected by the rotation of the femoral component (Supporting Information S1: Table 1). Three patients (1.4%) underwent RA‐TKA revision: one was subjected to debridement, antibiotic and implant retention (DAIR) procedure with exchange of the polyethylene insert for acute periprosthetic joint infection. One patient underwent implant removal and two‐stage revision for chronic periprosthetic joint infection, and one subject underwent open debridement and insert exchange for arthrofibrosis. No significant patello‐femoral complications were reported in any of the patients included in the study.

**Table 3:** Correlation between femoral component rotational alignment and patients' reported outcome measures.

	Correlation between outcomes and femoral component rotation (TEA)			
Outcome	Correlation	95% CI		*p* value
FJS‐12	0.11	−0.04	0.25	0.138
KOOS‐JR	0.04	−0.10	0.19	0.575

Abbreviations: CI, confidence interval; FJS‐12, Forgotten Joint Score‐12; KOOS‐JR, Knee Injury and Osteoarthritis Outcome Score for Joint Replacement; TEA, surgical transepicondylar axis.

## DISCUSSION

The most important finding of the present study was that, in the setting of patient‐specific FP, the rotational alignment of the femoral component changes significantly for different knee phenotypes, ranging from 6.9° internal rotation to 6.6° external rotation. However, this variability does not impact post‐operative clinical outcomes. Such variability in the axial alignment of the femoral component is directly associated with the differing coronal alignment of the tibial component among knee phenotypes (Table 2). This factor underscores the essence of patient‐specific FP, which embodies a multi‐dimensional strategy based on soft tissue tensioning and 3D bone morphology and provides a patient‐specific alternative to traditional alignment principles [19]. In this setting, although concerns about femoral component internal rotation with FP might raise, no significant patello‐femoral complications were reported in the study cohort. In addition, a direct significant association was identified between femoral component rotational alignment and tibial component coronal alignment. Last, the present study confirmed the lack of correlation between the coronal phenotypic alignment and the rotational alignment of the distal femur in the osteoarthritic knee [8, 12], underscoring the high variability of phenotypes both in the coronal and axial planes [12].

The implications of femoral component malrotation have been discussed extensively in the literature. Internal rotation of the femoral component has been associated with increased quadriceps requirements and alterations in tibio‐femoral kinematics [18, 25]. In addition, recent studies have also highlighted the increased risk of patellar instability when internal rotation is combined with excessive femoral component valgus [2, 13]. On the other hand, external rotation of the femoral component seems to enhance peak pressure by up to 35% in the lateral compartment and potentially reduce patello‐femoral contact stress [7]. Even in conventional TKA, targeting neutral MA and aiming for 3° of external rotation, femoral component rotation varies, ranging from 6.5° of internal to 6.5° of external rotation [3]. The findings of the present study support those by Moore et al., who reported on a series of image‐based RA‐TKA with FP and observed that femoral component rotational alignment approximates the TEA, with a negligible percentage of patello‐femoral complications [26]. The lower value of femoral component external rotation reported by Moore et al. in comparison to the data reported in this study can be explained by the larger boundary for tibial component coronal alignment (6° varus vs. 4° varus in this series). This finding justifies the counterintuitive direct association between tibial component coronal orientation and femoral component rotation and confirms that extending the boundary for tibial component coronal alignment, results in less femoral component external rotation to achieve flexion balance [28].

In conjunction with a low incidence of revision surgeries, data from the present study confirm that RA‐TKA with the FP technique does not result in significant patello‐femoral complications [9, 31]. This finding is even more meaningful in the setting where the mismatch between the anatomy of the patello‐femoral joint and current ‘off the shelf’ implants is well documented [11, 20, 29], with only 14.3% of the non‐osteoarthritic knees having parallel orientation of the anterior femoral joint line and the PCA [12].

In the literature, few studies have assessed patients' functional outcomes in relation to femoral component rotational alignment. Murgier et al. described clinical results of a patient cohort undergoing navigated CR‐TKA, with an ‘anatomical’ tibial cut up to 3° varus and the use of a ligament tensor to customize femoral component rotational alignment [27]. In concordance with the findings of the present study, they reported femoral axial alignment ranging from 7° internal to 8° external rotation relative to PCA, with no significant outcome differences with respect to femoral rotational alignment. Accordingly, others have reported that deviations from the 3° external rotation target in the setting of mechanically aligned TKA do not impact post‐operative clinical outcomes [3]. However, it is important to note that post‐operative PROMs are influenced by an array of factors beyond implant orientation. Preoperative pain levels, patient expectations, and the quality of post‐operative rehabilitation play crucial roles in determining PROM results. Therefore, while implant orientation is a significant consideration, it cannot solely account for the overall outcome of TKA.

The present study has several limitations, and results should therefore be interpreted with caution. First, due to the observational and retrospective study design, there is a potential risk of selection and recall bias. Second, as in most articles, the endpoint for determining patello‐femoral complications was the need for minor or major reoperations. This may underestimate the prevalence of patello‐femoral complaints that do not necessarily result in revision surgery, such as anterior knee pain without a detectable cause. Nevertheless, the reported good to excellent clinical outcomes suggest that this issue is unlikely to be significant. Third, knees were divided according to the CPAK phenotype classification by making assumptions to account for bone and cartilage wear.

Moreover, one notable limitation is the exclusive use of the CPAK classification for phenotypes, while other classifications were not employed. For instance, utilizing the functional knee phenotypes classification [14], could have led to different results in defining the classes and might have provided a more nuanced understanding of knee phenotypes, potentially revealing variations in femoral component rotation distribution that were not captured by the CPAK system. In addition, the CPAK classification was not derived from preoperative radiographs, but rather from preoperative CT scans. However, the measurement of femoral and tibial epiphyseal alignment using CT‐based robotic software is unaffected by lower limb positioning or knee fixed flexion deformity and has already been utilized in literature to describe coronal plane knee phenotypes [5, 8]. Furthermore, while femoral component rotation was considered only in relation to the TEA, other factors influencing patello‐femoral stability [15], such as patellar thickness and tracking, valgus alignment of the femoral component and trochlear groove morphology, were not evaluated. Another limitation of this study is the lack of preoperative PROMs, which prevents a thorough comparison of pre‐ and post‐operative patient outcomes. Finally, and most significantly, the soft tissue balancing and intraoperative planning procedures were influenced by the applied stress and left to the discretion of the surgeon, lacking standardization. The same knee could theoretically be balanced through various methods, such as adjusting the femoral or tibial alignment boundaries and setting different balancing targets. However, the force applied to balance the knee was not governed by flexion/extension gaps, and the FP alignment boundaries and balancing targets were consistent for all operating surgeons.

Moving forward, future clinical studies examining the FP strategy should attempt to focus on implant positioning and its effect on patello‐femoral tracking and patient‐reported outcome measures. The present study contributes to support the adoption of FP in TKA and highlights the efficacy of robotic‐assisted surgery in optimizing patello‐femoral and tibio‐femoral soft tissue balance, addressing patient‐specific phenotype differences.

## CONCLUSION

In the setting of image‐based RA‐TKA performed with the FP technique, the rotational alignment of the femoral component changes significantly among different knee phenotypes, ranging from 6.9° internal to 6.6° external rotation relative to the TEA. These variations do not affect clinical outcome at a minimum of 2 years of follow‐up and do not result in significant patello‐femoral complications.

## AUTHOR CONTRIBUTIONS

Generated the hypothesis, developed the study protocol and wrote the manuscript: Francesco Zambianchi. Developed the study protocol, collected and interpreted the data: Gabriele Bazzan. Collected primary data: Vincenzo Iorio. Analyzed the data: Riccardo Cuoghi Costantini. Collected primary data: Stefano Seracchioli. Performed data interpretation and revised the manuscript critically: Fabio Catani.

## CONFLICT OF INTEREST STATEMENT

The author, Francesco Zambianchi, reports speaking fees from Ab Medica S.p.A. The authors, Gabriele Bazzan, Stefano Seracchioli and Riccardo Cuoghi Costantini, or any member of their immediate family, have no funding or commercial associations (e.g. consultancies, stock ownership, equity interest and patent/licensing arrangements) that might pose a conflict of interest in connection with the submitted article. The author, Vincenzo Iorio, is a paid employee of Ab Medica S.p.A. The author, Fabio Catani, reports consultancy and speaking fees, royalties and fees for participation in review activities from Stryker.

## ETHICS STATEMENT

IRB approval was obtained by Comitato Etico Area Vasta Emilia Nord (7/2024/OSS*/AOUMO, transmitted with protocol no. 4527/2024). All patients signed and approved the General Informed Consent prior to surgery and consented to the retrospective use of intraoperative and post‐operative data.

## Supporting information

Supporting information.

## Data Availability

The data that support the findings of this study are available from the corresponding author upon reasonable request.

## References

[1] Akagi M , Oh M , Nonaka T , Tsujimoto H , Asano T , Hamanishi C . An anteroposterior axis of the tibia for total knee arthroplasty. Clin Orthop Relat Res. 2004;420(420):213–219. 10.1097/00003086-200403000-00030 15057100

[2] Assiotis A , To K , Morgan‐Jones R , Pengas IP , Khan W . Patellar complications following total knee arthroplasty: a review of the current literature. Eur J Orthop Surg Traumatol. 2019;29(8):1605–1615. 31302764 10.1007/s00590-019-02499-z

[3] Becker R , Bäker K , Hommel H , Bernard M , Kopf S . No correlation between rotation of femoral components in the transverse plane and clinical outcome after total knee arthroplasty. Knee Surg Sports Traumatol Arthrosc. 2019;27(5):1456–1462. 29767270 10.1007/s00167-018-4981-8

[4] Behrend H , Giesinger K , Giesinger JM , Kuster MS . The “Forgotten Joint” as the ultimate goal in joint arthroplasty. J Arthroplasty. 2012;27(3):430–436.e1. 22000572 10.1016/j.arth.2011.06.035

[5] Bertugli E , Zambianchi F , Batailler C , Bazzan G , Lustig S , Catani F . Change of CPAK class does not affect functional outcomes in robotic arm‐assisted total knee arthroplasty performed with functional alignment. Knee Surg Sports Traumatol Arthrosc. 2025;33(5):1773–1783. 39666596 10.1002/ksa.12561

[6] Cassard X , Garnault V , Corin B , Claverie D , Murgier J . Outpatient total knee arthroplasty: readmission and complication rates on day 30 in 61 patients. Orthop Traumatol Surg Res. 2018;104(7):967–970. 30179723 10.1016/j.otsr.2018.07.014

[7] Chen Z , Wang L , Liu Y , He J , Lian Q , Li D , et al. Effect of component mal‐rotation on knee loading in total knee arthroplasty using multi‐body dynamics modeling under a simulated walking gait. J Orthop Res. 2015;33(9):1287–1296. 25820991 10.1002/jor.22908

[8] Corbett J , Sinha P , Esposito CI , Wood JA , Chen DB , MacDessi SJ . Multi‐planar expansion of the coronal plane alignment of the knee classification? A computed tomographic study indicates no significant correlation with alignment parameters in other planes. J Arthroplasty. 2024;39(2):336–342. 37586596 10.1016/j.arth.2023.08.033

[9] Genestoux V , Vermorel PH , Neri T , Farizon F , Philippot R . Does inverse kinematic alignment coupled with robot‐assisted TKA optimize patellofemoral clinical and radiological results? Orthop Traumatol Surg Res. 2024;110:103880. 38582224 10.1016/j.otsr.2024.103880

[10] Gregori P , Koutserimpas C , Giovanoulis V , Batailler C , Servien E , Lustig S . Functional alignment in robotic‐assisted total knee arthroplasty for valgus deformity achieves safe coronal alignment and excellent short‐term outcomes. Knee Surg Sports Traumatol Arthrosc. 2025;33:2187–2196. 39821487 10.1002/ksa.12585 PMC12104782

[11] Hazratwala K , O'Callaghan WB , Dhariwal S , Wilkinson MPR . Wide variation in tibial slopes and trochlear angles in the arthritic knee: a CT evaluation of 4116 pre‐operative knees. Knee Surg Sports Traumatol Arthrosc. 2022;30(9):3049–3060. 34487188 10.1007/s00167-021-06725-2

[12] Hess S , Chelli S , Leclercq V , Lustig S , Graichen H , Hirschmann MT . Three‐compartment phenotype concept of total knee arthroplasty alignment: mismatch between distal femoral, posterior femoral, and tibial joint lines. J Arthroplasty. 2025. In press. 10.1016/j.arth.2025.02.015 40049560

[13] Heyse TJ , El‐Zayat BF , De Corte R , Chevalier Y , Fuchs‐Winkelmann S , Labey L . Internal femoral component malrotation in TKA significantly alters tibiofemoral kinematics. Knee Surg Sports Traumatol Arthrosc. 2018;26(6):1767–1775. 29128876 10.1007/s00167-017-4778-1

[14] Hirschmann MT , Moser LB , Amsler F , Behrend H , Leclerq V , Hess S . Functional knee phenotypes: a novel classification for phenotyping the coronal lower limb alignment based on the native alignment in young non‐osteoarthritic patients. Knee Surg Sports Traumatol Arthrosc. 2019;27(5):1394–1402. 30976825 10.1007/s00167-019-05509-z

[15] Howell SM , Gill M , Shelton TJ , Nedopil AJ . Reoperations are few and confined to the most valgus phenotypes 4 years after unrestricted calipered kinematically aligned TKA. Knee Surg Sports Traumatol Arthrosc. 2022;30(3):948–957. 33582829 10.1007/s00167-021-06473-3 PMC8901497

[16] Itou J , Gazali I , Pandit H , Okazaki K , Ascani D , Peersman G . The CPAK classification in three‐dimensional measurements is consistent with those in two‐dimensional measurements. Arch Orthop Trauma Surg. 2025;145(1):160. 39932590 10.1007/s00402-024-05742-3

[17] Kafelov M , Batailler C , Shatrov J , Al‐Jufaili J , Farhat J , Servien E , et al. Functional positioning principles for image‐based robotic‐assisted TKA achieved a higher Forgotten Joint Score at 1 year compared to conventional TKA with restricted kinematic alignment. Knee Surg Sports Traumatol Arthrosc. 2023;31(12):5591–5602. 37851026 10.1007/s00167-023-07609-3

[18] Klasan A , de Steiger R , Holland S , Hatton A , Vertullo CJ , Young SW . Similar risk of revision after kinematically aligned, patient‐specific instrumented total knee arthroplasty, and all other total knee arthroplasty: combined results from the Australian and New Zealand Joint Replacement Registries. J Arthroplasty. 2020;35(10):2872–2877. 32620297 10.1016/j.arth.2020.05.065

[19] Koutserimpas C , Andriollo L , Gregori P , Zambianchi F , Tsiridis E , Catani F , et al. Revisiting terminology: the transition from “functional alignment” to “functional knee positioning”. Knee Surg Sports Traumatol Arthrosc. 2025;33(6):1948–1953. 40167115 10.1002/ksa.12667

[20] Kuo AW , Chen DB , Wood J , MacDessi SJ . Modern total knee arthroplasty designs do not reliably replicate anterior femoral morphology. Knee Surg Sports Traumatol Arthrosc. 2020;28(9):2808–2815. 31352496 10.1007/s00167-019-05610-3

[21] Luyckx T , Moreels R , Geernaert H , Scheys L , Vandenneucker H . Valgus alignment of the femoral component is associated with higher revision rates 10 years after TKA. Knee Surg Sports Traumatol Arthrosc. 2023;31(10):4171–4178. 37154911 10.1007/s00167-023-07448-2

[22] Lyman S , Lee YY , Franklin PD , Li W , Cross MB , Padgett DE . Validation of the KOOS, JR: a short‐form knee arthroplasty outcomes survey. Clin Orthop Relat Res. 2016;474(6):1461–1471. 26926773 10.1007/s11999-016-4719-1 PMC4868168

[23] MacDessi SJ , Griffiths‐Jones W , Harris IA , Bellemans J , Chen DB . Coronal Plane Alignment of the Knee (CPAK) classification: a new system for describing knee phenotypes. Bone Joint J. 2021;103‐B(2):329–337. 10.1302/0301-620X.103B2.BJJ-2020-1050.R1 PMC7954147 33517740

[24] Mancino F , Fontalis A , Kayani B , Magan A , Plastow R , Haddad FS . The current role of CT in total knee arthroplasty. Bone Joint J. 2024;106‐B(9):892–897. 10.1302/0301-620X.106B9.BJJ-2023-1303.R1 39216858

[25] Manning WA , Ghosh KM , Blain A , Longstaff L , Rushton SP , Deehan DJ . Internal femoral component rotation adversely influences load transfer in total knee arthroplasty: a cadaveric navigated study using the Verasense device. Knee Surg Sports Traumatol Arthrosc. 2018;26(5):1577–1585. 28712028 10.1007/s00167-017-4640-5 PMC5907629

[26] Moore J , Van de Graaf VA , Wood JA , Chen DB , MacDessi SJ . In functionally aligned total knee arthroplasty, femoral component rotation follows the transepicondylar axis to achieve flexion balance. Knee Surg Sports Traumatol Arthrosc. 2025;33(5):1784–1791. 39838919 10.1002/ksa.12590

[27] Murgier J , Clatworthy M . Variable rotation of the femur does not affect outcome with patient specific alignment navigated balanced TKA. Knee Surg Sports Traumatol Arthrosc. 2022;30(2):517–526. 32783080 10.1007/s00167-020-06226-8

[28] Ollivier B , Wakelin E , Plaskos C , Vandenneucker H , Luyckx T . Widening of tibial resection boundaries increases the rate of femoral component valgus and internal rotation in functionally aligned TKA. Knee Surg Sports Traumatol Arthrosc. 2024;32(4):953–962. 38444096 10.1002/ksa.12118

[29] Rosa SB , Hazratwala K , Wilkinson MPR . Mismatch between trochlear coronal alignment of arthritic knees and currently available prosthesis: a morphological analysis of 4116 knees and 45 implant designs. Knee Surg Sports Traumatol Arthrosc. 2023;31(8):3116–3123. 36456826 10.1007/s00167-022-07251-5 PMC10356655

[30] Shatrov J , Batailler C , Sappey‐Marinier E , Gunst S , Servien E , Lustig S . Kinematic alignment fails to achieve balancing in 50% of varus knees and resects more bone compared to functional alignment. Knee Surg Sports Traumatol Arthrosc. 2022;30(9):2991–2999. 35962840 10.1007/s00167-022-07073-5

[31] Shatrov J , Coulin B , Batailler C , Servien E , Walter B , Lustig S . Alignment philosophy influences trochlea recreation in total knee arthroplasty: a comparative study using image‐based robotic technology. Int Orthop. 2023;47(2):329–341. 36112197 10.1007/s00264-022-05570-3 PMC9877070

[32] Spitzer A , Gorab R , Barrett W , Nassif N , Hunter M , Leslie I , et al. Robotic‐assisted total knee arthroplasty reduces soft‐tissue releases which improves functional outcomes: a retrospective study. Knee. 2024;49:52–61. 38848658 10.1016/j.knee.2024.05.008

[33] Twiggs JG , Dickison DM , Kolos EC , Wilcox CE , Roe JP , Fritsch BA , et al. Patient variation limits use of fixed references for femoral rotation component alignment in total knee arthroplasty. J Arthroplasty. 2018;33(1):67–74. 28927560 10.1016/j.arth.2017.08.023

[34] Winnock de Grave P , Luyckx T , Van Criekinge T , Müller JH , Ollivier B , Van Eecke E , et al. Inverse kinematic alignment accommodates native coronal knee alignment better in comparison to adjusted mechanical alignment and restricted kinematic alignment. Knee Surg Sports Traumatol Arthrosc. 2023;31(9):3765–3774. 36781450 10.1007/s00167-023-07326-x

[35] Zambianchi F , Bazzan G , Marcovigi A , Pavesi M , Illuminati A , Ensini A , et al. Joint line is restored in robotic‐arm‐assisted total knee arthroplasty performed with a tibia‐based functional alignment. Arch Orthop Trauma Surg. 2021;141(12):2175–2184. 34255176 10.1007/s00402-021-04039-z

[36] Zambianchi F , Matveitchouk N , Pavesi M , Clemenza S , Cuoghi Costantini R , Marcovigi A , et al. Small deviations between planned and performed bone cuts using a CT‐based robotic‐arm‐assisted total knee arthroplasty system. Knee Surg Sports Traumatol Arthrosc. 2024;32(6):1539–1547. 38572678 10.1002/ksa.12171

