# Supplementary material for: Femoral component rotational alignment in robotic‐assisted total knee arthroplasty with functional knee positioning varies across knee phenotypes without affecting clinical outcomes
Source: Knee Surg Sports Traumatol Arthrosc. 2025 Jun 29;33(11):3915–24. doi: 10.1002/ksa.12732 (PMC12582239; doi:10.1002/ksa.12732)
Supplement: Supplementary file 1 — Supporting information. [file KSA-33-3915-s001.docx]

**Supplementary Material**

**Table 1**

Association between femoral component rotational alignment and patients’ reported outcome measures expressed for the different preoperative CPAK classes. TEA: surgical transepicondylar axis; FJS-12: Forgotten Joint Score-12; KOOS-JR: Knee Injury and Osteoarthritis Outcome Score for Joint Replacement.

|  |  | Correlation with femoral component rotation (TEA) | | |  |
| --- | --- | --- | --- | --- | --- |
| CPAK | Outcome | Estimate | 95% CI | | p-value |
| I | FJS-12 | 0.19 | -0.07 | 0.43 | 0.151 |
| I | KOOS-JR | 0.19 | -0.07 | 0.43 | 0.157 |
| II | FJS-12 | 0.14 | -0.15 | 0.41 | 0.346 |
| II | KOOS-JR | -0.09 | -0.37 | 0.20 | 0.559 |
| III | FJS-12 | -0.20 | -0.50 | 0.13 | 0.230 |
| III | KOOS-JR | -0.06 | -0.38 | 0.27 | 0.729 |
| IV | FJS-12 | 0.09 | -0.39 | 0.54 | 0.710 |
| IV | KOOS-JR | 0.01 | -0.46 | 0.47 | 0.970 |
| V | FJS-12 | 0.37 | -0.20 | 0.75 | 0.190 |
| V | KOOS-JR | 0.43 | -0.13 | 0.78 | 0.122 |
| VI | FJS-12 | -0.11 | -0.80 | 0.70 | 0.819 |
| VI | KOOS-JR | -0.15 | -0.81 | 0.68 | 0.755 |

**Table 2**

Differences between mean femoral component rotational alignment among different preoperative CPAK classes expressed in terms of p-values.

| CPAK | I | II | III | IV | V | VI |
| --- | --- | --- | --- | --- | --- | --- |
| I | - | 0.026 | 0.000 | 0.514 | 0.149 | 0.000 |
| II | 0.026 | - | 0.003 | 1.000 | 1.000 | 0.053 |
| III | 0.000 | 0.003 | - | 0.015 | 0.186 | 1.000 |
| IV | 0.514 | 1.000 | 0.015 | - | 1.000 | 0.054 |
| V | 0.149 | 1.000 | 0.186 | 1.000 | - | 0.202 |
| VI | 0.000 | 0.053 | 1.000 | 0.054 | 0.202 | - |
